# Case of Mania in a Child Six Years Old

**Published:** 1848-04-01

**Authors:** T. C. Morison

**Affiliations:** SURGEON


### CASE OF MANIA OCCURRING IN A CHILD SIX YEARS OLD.

BY T. C. MORISON, ESQ., SURGEON.

THE appearance of insanity before the age of puberty is at all times a circumstance of so rare occurrence, as to render it highly important that all such cases should be properly recorded and authenticated.

Although various instances are related of children of the ages of twelve, nine, and eight years, being afflicted with mania, nostalgia, and even jealousy, amounting to such an extent as to verge upon partial insanity, I have never met with any recorded case in which the disorder made its appearance at so early an age as it did in the following case. The unsoundness of intellect in children is, in general, idiocy; cases of which, with a disposition to violence and mischief, are to be met with in most large asylums at a very early age. The phenomena presented in such cases, however, are of a totally different nature, and owe their origin to totally different causes.

E. A., aged six years, the daughter of an old furniture broker, was admitted into Bethlehem Hospital on the 30th August, 1842, labouring under an attack of mania, of ten weeks' duration. Her education was good for her years, and she never, in any way, gave her friends reason to suppose that she was deficient in intellect. There did not exist any hereditary tendency to insanity or to epilepsy in the family. The immediate cause of the attack was stated to have been inflammation of the brain, preceded by a fit of convulsions. From her previous history, it appeared that since the age of eighteen months, she had been subject to the occasional recurrence of these fits; that they first made their appearance in consequence of teething, and were of rather a severe nature.

Immediately before her admission, she had been under treatment at St. George's Hospital for inflammation of the brain. At the time of her admission into that hospital, the following is the report of her case—" Attacked on June 3rd with convulsions; had a similar attack at the age of eighteen months, when teething, and has twice been similarly seized; appeared in very good health previously to the present illness; is now wholly unconscious; is, in fact, in a state of coma."

When admitted into Bethlehem Hospital, her conduct was violent and mischievous; striking those about her, tearing her clothes, and destroying everything within her reach. She was generally incoherent in her speech —repeating any words she might hear in a monotonous voice, and without appearing to understand them, such as " Poor thing—poor thing !" Occasionally, however, by strongly arresting her attention, a correct reply could be obtained from her. The expression of her countenance was quick and animated; her general health was good, and she ate and slept well. Sixteen days after her admission, she was attacked with diarrhœa of a mild character, from which she recovered at the end of a few days. Soon afterwards, a considerable improvement took place in her general behaviour, and she began to pay attention to the directions of one of the convalescent patients, who took charge of her; would say, "Thank you, sir," on receiving any little present, and make a courtesy, &c. She also gave over many of her mischievous tricks; but still continued decidedly insane. She could not be induced to employ herself in any way, and was subject to violent and unaccountable outbursts of passion, in which she tore her clothes, and bit and scratched all who attempted to restrain her.

After she had remained about six months in the hospital, she became much more docile, and began to employ herself in sewing, &c. From this time, also, a marked improvement gradually took place in her manner and conduct, until she was reported well, after having been about twenty months under treatment. She was, however, allowed to remain in the hospital for about six months longer, until she could be transmitted to the care of her friends, who were all abroad. At this time she appeared to be a modest, quiet, and intelligent looking girl.

